# WAFNRLTG: A Novel Model for Predicting LncRNA Target Genes Based on Weighted Average Fusion Network Representation Learning Method

**DOI:** 10.3389/fcell.2021.820342

**Published:** 2022-01-19

**Authors:** Jianwei Li, Zhenwu Yang, Duanyang Wang, Zhiguang Li

**Affiliations:** ^1^ School of Artificial Intelligence, Institute of Computational Medicine, Hebei University of Technology, Tianjin, China; ^2^ Hebei Province Key Laboratory of Big Data Calculation, Hebei University of Technology, Tianjin, China

**Keywords:** lncRNA target genes prediction, weighted average fusion network representation learning, heterogeneous network, machine learning, XGBoost

## Abstract

Long non-coding RNAs (lncRNAs) do not encode proteins, yet they have been well established to be involved in complex regulatory functions, and lncRNA regulatory dysfunction can lead to a variety of human complex diseases. LncRNAs mostly exert their functions by regulating the expressions of target genes, and accurate prediction of potential lncRNA target genes would be helpful to further understanding the functional annotations of lncRNAs. Considering the limitations in traditional computational methods for predicting lncRNA target genes, a novel model which was named Weighted Average Fusion Network Representation learning for predicting LncRNA Target Genes (WAFNRLTG) was proposed. First, a novel heterogeneous network was constructed by integrating lncRNA sequence similarity network, mRNA sequence similarity network, lncRNA-mRNA interaction network, lncRNA-miRNA interaction network and mRNA-miRNA interaction network. Next, four popular network representation learning methods were utilized to gain the representation vectors of lncRNA and mRNA nodes. Then, the representations of lncRNAs and target genes in the heterogeneous network were obtained with the weighted average fusion network representation learning method. Finally, we merged the representations of lncRNAs and related target genes to form lncRNA-gene pairs, trained the XGBoost classifier and predicted potential lncRNA target genes. In five-cross validations on the training and independent datasets, the experimental results demonstrated that WAFNRLTG obtained better AUC scores (0.9410, 0.9350) and AUPR scores (0.9391, 0.9350). Moreover, case studies of three common lncRNAs were performed for predicting their potential lncRNA target genes and the results confirmed the effectiveness of WAFNRLTG. The source codes and all data of WAFNRLTG can be freely downloaded at https://github.com/HGDYZW/WAFNRLTG.

## Introduction

Long non-coding RNAs (lncRNAs) are important components of non-coding RNAs whose transcript lengths exceed 200 nucleotides (Ponting et al., 2009). LncRNAs generally exhibit low cross-species conservation, low expression levels and high tissue specificity (Mercer et al., 2008; Pauli et al., 2012), and do not have the functions of protein coding (Carninci and Hayashizaki, 2007). LncRNAs can interfere with the expressions of downstream genes through base complementary pairing, and participate in most biological processes, including cell proliferation, differentiation, chromatin remodeling, epigenetic regulation, genomic splicing, transcription, translation and other aspects (Lander et al., 2001; Guttman et al., 2009; Mercer et al., 2009; Wapinski and Chang, 2011). Due to the important role of lncRNAs in biological processes, their regulatory dysfunctions are commonly associated with a variety of human diseases, especially cancers (Gupta et al., 2010; Zhang et al., 2016). Recent studies have found that lncRNAs regulate many key biological processes by interacting with their target genes. For example, the binding of lncRNA BACE1-AS with its target gene BACE1 increases the stability of BACE1, which regulates BACE1 profile and subsequently affects BACE1 protein expression (Faghihi et al., 2008). In addition, lncRNAs can also be used as the competing endogenous RNA to indirectly regulate mRNA through the shared miRNAs. For example, lncRNA HULC can competitively regulate PRKACB by sharing the common binding site of miR-372, and induce the phosphorylation of CREB in liver cancer (Qi et al., 2015). Since lncRNAs have an important role in biological processes, it prompted researchers to develop computational methods to identify lncRNA regulatory functions. Currently, these computational methods are mainly classified into two categories based on their aims, in terms of lncRNA related diseases identification and lncRNA target genes identification.

In computational models aimed on diseases identification, they can be further divided into two groups: machine learning methods and biological network methods. In recent years, machine learning has been widely applied to predict lncRNA-disease associations. These methods extract the biological features of lncRNAs and diseases and then use machine learning classifiers to infer lncRNA-related diseases. Chen et al. (Chen and Yan, 2013) developed a novel model, LRLSLDA, which predicted potential disease-related lncRNAs in a semi-supervised learning framework. In addition, LRLSLDA requires only human lncRNA expression profiles and known lncRNA-disease associations without negative samples to produce reliable results. Lan et al. (2017) proposed LDAP model to predict lncRNA-disease associations by using a bagging SVM classifier based on lncRNA similarity and disease similarity. Yao et al. (2020) implemented a random forest and feature selection-based lncRNA-disease association prediction model, RFLDA. RFLDA integrated experimentally supported miRNA-disease associations, lncRNA-disease associations, disease semantic similarity, lncRNA functional similarity and lncRNA-miRNA interactions as input features. RFLDA selected the most useful features to train the prediction model by feature selection based on the importance scores of random forest variables. Based on the hypothesis that similar diseases are more likely to be associated with similar lncRNAs, a number of biological network-based lncRNA-disease association prediction methods have been proposed. Sun et al. (2014) proposed a global network-based computational method named RWRlncD by integrating disease similarity network, lncRNAs functional similarity network and known lncRNA-disease associations. Zhou et al. (2015) proposed RWRHLD method to predict lncRNA-disease associations, which integrated three networks into a heterogeneous network and implemented a random walk on it. Deng et al. (2021) came up with a method, LDAH2V, for inferring lncRNA-disease associations by integrating lncRNA-disease associations, miRNA co-expression profiles, miRNA-disease associations, lncRNA-miRNA associations and lncRNA functional similarity. LDAH2V is a generic network-based link prediction model that can be applied to any number of entity networks.

The theoretical foundation for lncRNAs target genes prediction is the assumption that highly similar lncRNAs tend to have similar interaction. Many studies have shown that lncRNAs indirectly regulate gene expressions via adjusting expressions of miRNAs (Jones-Rhoades and Bartel, 2004). Therefore, exploring the interaction of lncRNA-miRNA would contribute to understand the complex functions of lncRNAs. Due to the rapid development of RNA sequencing technology, lncRNA-related and miRNA-related biological data are increasing rapidly. Predicting the interactions between lncRNAs and miRNAs through traditional experimental methods is very time-consuming and labor-intensive. Recently, many computational methods for predicting lncRNA-miRNA interactions have been proposed. For example, Wong et al. (2020) proposed the LNRLMI model, which constructed a bipartite network to predict potential lncRNA-miRNA interactions by combining the known interaction network and the similarity of the expression profile of lncRNA-miRNA. Zhou et al. (2019) proposed a GEEL model that constructed the lncRNA-miRNA interaction network based on the sequence features and known interactions of lncRNA and miRNA, and then used five different graph embedding methods to obtain the node representation of lncRNA/miRNA. Based on the embedding results, GEEL used individual graph embedding method-based model as basic predictors and build an ensemble model to predict the potential interactions between lncRNAs and miRNAs. Yang et al. (2020) proposed the lncMirNet model which predicted lncRNA-miRNA interactions based on hybrid sequence features. Based on these, Zhao et al. (2020) developed a method, named DeepLGP, for prioritizing lncRNA target genes via encoding gene and lncRNA features. These features were used by the convolutional neural network and were combined as the features of lncRNA-gene pairs. Finally, the CNN model was used to classify lncRNA-gene pairs into true or false pairs.

**FIGURE 1:**
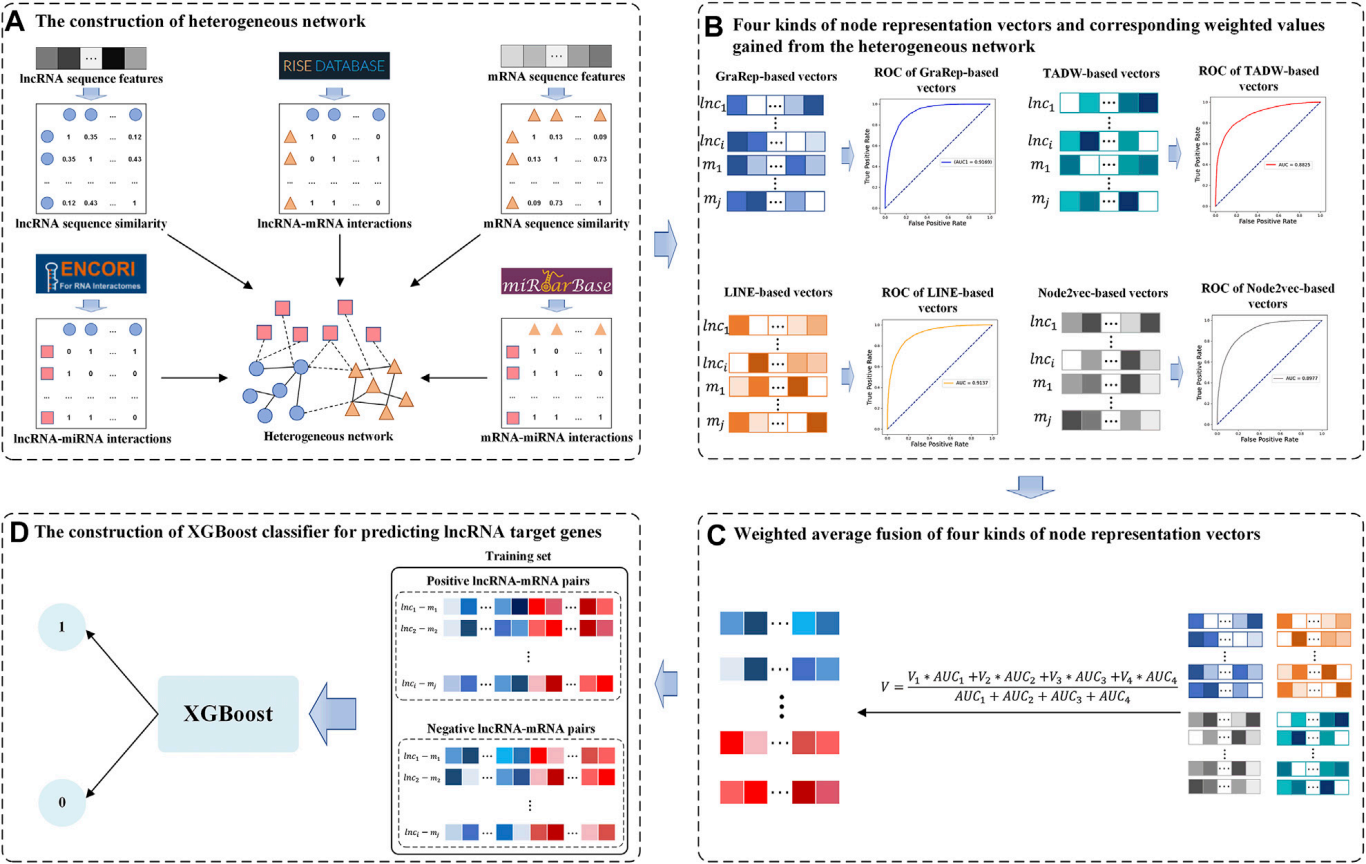
Flow chart of WAFNRLTG. **(A)** Two sequence similar networks (lncRNA-lncRNA, mRNA-mRNA) are constructed based on the sequence features of lncRNA/mRNA and three interaction networks (lncRNA-mRNA, lncRNA-miRNA, mRNA-miRNA) are constructed based on various data sources. The heterogeneous network is established with the above five networks. **(B)** Four kinds of network representation learning methods are employed to gain node representation vectors of lncRNAs and mRNAs from the heterogeneous network. Based on these vectors, the AUC scores of the corresponding models are gained as weighted values. **(C)** The new node representation vectors are gained via fusing the four kinds of representation vectors with the weighted average method **(D)** The representation vectors of lncRNAs and mRNAs are combined to train the XGBoost classifier for predicting the target genes of lncRNAs.

In this study, we developed a Weighted Average Fusion Network Representation Learning method-based model to improve the performance of lncRNA Target Genes prediction (WAFNRLTG). First, we constructed a heterogeneous network, which integrated two similar networks and three interaction networks. Next, the network representation learning method was utilized to gain the representation vectors of lncRNA and mRNA nodes. Four popular network representation learning methods (GraRep, LINE, TADW, and Node2vec) were adopted in our model. Then, the weighted average method was further employed to fuse the corresponding representation vectors according to the AUC scores. The novel representation vectors were obtained which integrated different network structure information and improved the generalization ability of the model. Finally, the representation vectors of lncRNAs and the representation vectors of mRNAs were merged to form the lncRNA-gene pairs, and XGBoost classifier was built based on the merged representations of lncRNA-miRNA pairs. With five-fold cross-validations on training and independent dataset, the experimental results demonstrated that WAFNRLTG obtained AUC scores (0.9410, 0.9350) and AUPR scores (0.9391, 0.9350), and outperformed the individual network representation learning method-based models. Furthermore, three case studies were performed to evaluate the capability of WAFNRLTG. The results can be verified by the existing PubMed literatures. In conclusion, WAFNRLTG is an effective tool for predicting the potential lncRNA target genes. The flow chart of WAFNRLTG is shown in Figure 1.

## Materials and Methods

### Datasets

In this paper, there were 5,435 validated lncRNA-gene interactions used as positive samples which were obtained from the RISE database (Hubbard et al., 2002). Negative samples were randomly selected from all unknown lncRNA-gene interactions. Because the number of unknown lncRNA-gene interactions are far more than the number of positive samples, a total of 5,435 negative samples were generated with the same number of positive samples. Eventually, we obtained 5,435 positive samples and 5,435 negative samples as a new dataset. Subsequently, five-sixth of the new dataset were randomly selected as training samples to train the classifier, and the remaining samples were used as the independent samples to evaluate each classifier.

### LncRNA and mRNA Sequence Similarity Networks

**FIGURE 2:**
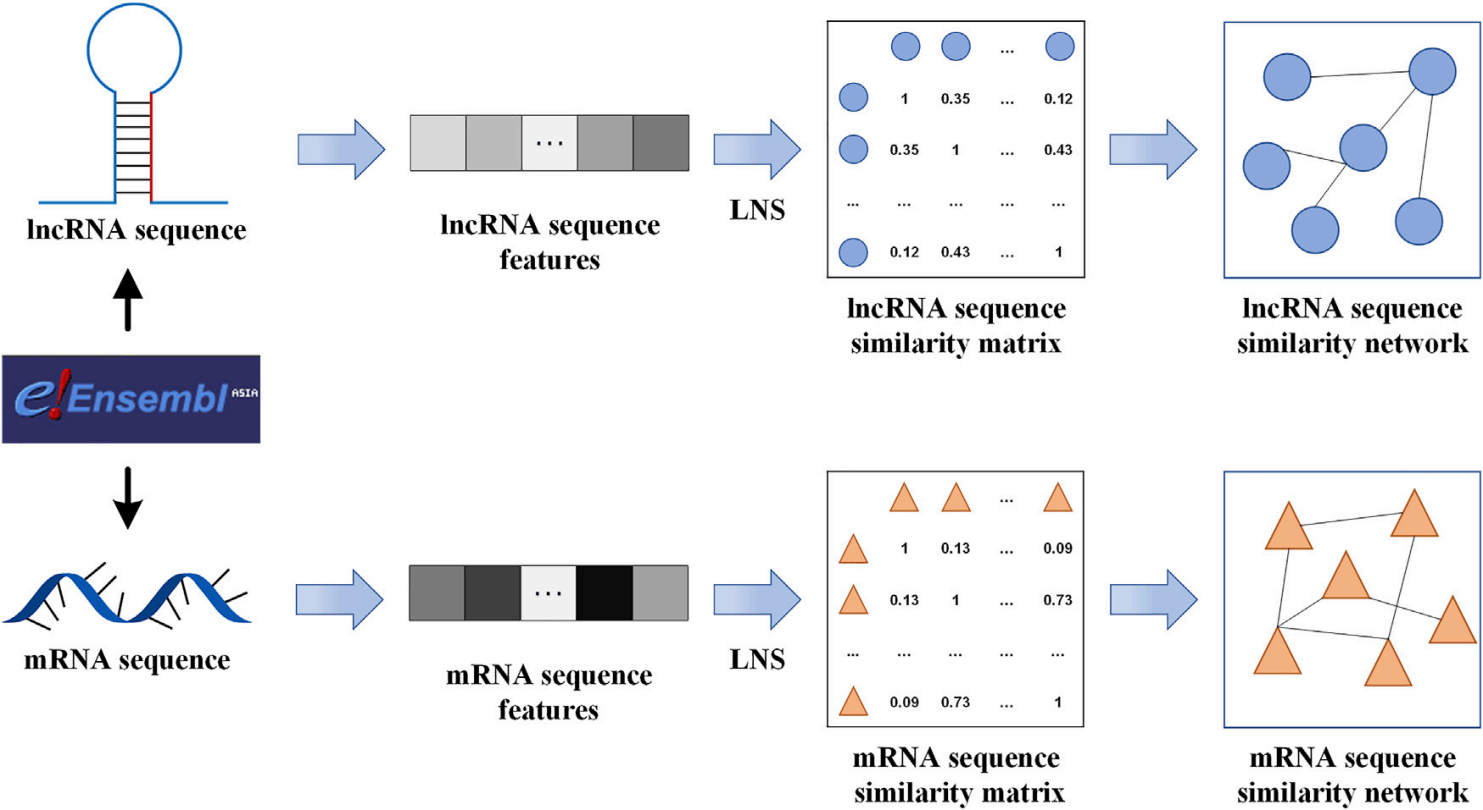
The flowchart of constructing the lncRNA and mRNA sequence similarity network.

The corresponding sequences of lncRNAs and mRNAs were downloaded from the Ensembl database (Hubbard et al., 2002) according to their corresponding Ensembl IDs. Linear neighborhood similarity measure (LNS) and two lncRNA (miRNA) sequence features [k-mer (Gupta et al., 2008) and CTD (Tong and Liu, 2019)] were employed to calculate the lncRNA similarities and miRNA similarities respectively. For each RNA sequence, its k-mer frequency distribution is usually defined as the occurrence frequency of corresponding k-length contiguous subsequences. LNS is a recently proposed similarity calculation method and has been widely used in the field of bioinformatics. The 30-dimensional CTD (composition, transition, and distribution) features are used to represented RNA structure information. In order to construct lncRNA and mRNA sequence similarity networks, the 3-mers features and CTD features of a lncRNA/mRNA sequence were merged into union vectors respectively. For the lncRNA sequence similarity network, the union vectors were used to construct the lncRNA similarity matrix by LNS (Zhang et al., 2017). For example, given a specified lncRNA, its top ten lncRNAs with similarity weights greater than 0 were considered to be linked with it. Based on this strategy, closely homologous lncRNAs were linked with this lncRNA for establishing the lncRNA sequence similarity network. For the mRNA sequence similarity network, its construction procedure was the same as those of the lncRNA sequence similarity network. After this, we gained a lncRNA sequence similarity network involving 2,249 lncRNAs and mRNA sequence similarity network involving 3,785 mRNAs. The flowchart of constructing the lncRNA and mRNA sequence similarity network is shown in Figure 2.

### LncRNA-mRNA Interaction Network

In this study, experimentally validated lncRNA-mRNA interaction data was downloaded from the RISE database (Gong et al., 2018), and it included 10,941 lncRNA-mRNA interactions. After removing the redundant data and nonhuman data, 5,435 associations involving 2,249 lncRNAs and 3,785 mRNAs were finally obtained. Therefore, the lncRNA-mRNA interaction network in our model was constructed based on these 5,435 interactions.

### LncRNA-miRNA Interaction Network

First, the known lncRNA-miRNA interactions were downloaded from the ENCORI database (Li et al., 2014). Then, the duplicate interactions were removed and the only interactions between the lncRNAs which were from the lncRNA-mRNA interactions and miRNAs were preserved. In the end, the lncRNA-miRNA interaction network was constructed with 6,053 lnRNA-miRNA interactions between 2,249 lncRNAs and 636 miRNAs.

### MRNA-miRNA Interaction Network

We downloaded the known mRNA-miRNA interactions from the miTarbese database (Huang et al., 2020). Then, the duplicate interactions were removed and the only interactions between the mRNAs which were from the lncRNA-mRNA interactions and the miRNAs which were from the lncRNA-miRNA interactions were retained. Ultimately, the constructed mRNA-miRNA network in our study contained 1983 mRNA-miRNA interactions between 305 mRNAs and 636 miRNAs.

### Network Representation Learning of the Heterogeneous Network

Recently, many Network Representation Learning (NRL) methods have been proposed (Zhang et al., 2020), of which main purpose is to find a proper mapping function to map large-scale, high-dimensional, sparse vectors into a low-dimensional, dense semantic space, while keep the proximity of these low-dimensional vector representations to the original network. NRL has attracted the attention of scholars in the fields of data mining of biological information data. The low-dimensional representation learned from the network representation is applied to downstream network analysis tasks, such as node classification (Tang et al., 2016a), link prediction, association mining (Zhao et al., 2019), information recommendation (Han et al., 2018) and network visualization (Tang et al., 2016b).

In our study, four state-of-the-art network representation learning methods [GraRep (Cao et al., 2015), LINE (Tang et al., 2015), TADW (Yang et al., 2015) and Node2vec (Grover and Leskovec, 2016)] were used to learn the representation vectors of lncRNA nodes and mRNA nodes for making full use of the various useful information in the heterogeneous network.

LINE maintains both first-order and second-order proximity during learning node representations. Given an undirected edge (i, j) , the joint probability of node $v_{i}$ and node $\;v_{j}$ is as follows:

$$p_{1}\left(v_{i},v_{j}\right)=\frac{1}{1+\exp\left(-{\vec{u}}_{i}^{T}\cdot {\vec{u}}_{j}\right)}$$ (1)

where ${\vec{u}}_{j}\in \;R^{d}$ is the low-dimensional vector representation of node $v_{i}$ . The empirical probability of the distribution p(⋅,⋅) on the space V×V is:

$${\hat{p}}_{i}\left(i,j\right)=\frac{w_{ij}}{W}$$ (2)

where $W=\sum_{\left(i,j\right)\in E}w_{ij}$ and $w_{ij}$ is the weight of edge (i,j) .

After optimizing the model by minimizing the KL scatter of the two distributions, the objective function is defined as follows:

$$O_{1}=-\sum_{\left(i,j\right)\in E}w_{ij}\log p_{1}\left(v_{i},v_{j}\right)$$ (3)

The second-order similarity scenario assumes that nodes sharing a large number of connections with other nodes are similar to each other, and each node is considered as a specific context, then nodes with similar distribution on the context are similar. Here, two vectors ${\vec{u}}_{i}$ and ${\vec{u}}_{i}^{'}$ are introduced, where ${\vec{u}}_{i}$ is the representation of $v_{i}$ when it is treated as a vertex and ${\vec{u}}_{i}^{'}$ is the representation of $v_{i}$ when it is treated as a specific “context”. For an undirected edge (i,j) , the probability of generating the context $v_{j}$ from $v_{i}$ is:

$$p_{2}\left(v_{j}|v_{i}\right)=\frac{\exp\left({\vec{u}}_{j}^{\prime T}\cdot {\vec{u}}_{i}\right)}{\sum_{k=1}^{\left|V\right|}\exp\left({\vec{u}}_{k}^{\prime T}\cdot {\vec{u}}_{i}\right)}$$ (4)

where |V| denotes the number of nodes or contexts. The empirical distribution ${\hat{p}}_{2}\left(\cdot |v_{i}\right)$ is defined as follows:

$${\hat{p}}_{2}\left(v_{j},v_{i}\right)=\frac{w_{ij}}{d_{i}}$$ (5)

where $w_{ij}$ is the weight of edge (i,j) and $d_{i}$ is the out-degree of node $v_{i}$ . $d_{i}$ is used in LINE as the importance of nodes $\lambda_{i}$ . By using the KL scatter while ignoring some constants, the objective function is obtained as follows:

$$O_{2}=-\sum_{\left(i,j\right)\in E}w_{ij}\log p_{2}\left(v_{j},v_{i}\right)$$ (6)

LINE also adopted negative sampling to optimize the model, while using the Alias method to accelerate the sampling process.

GraRep extends LINE by learning the k-order relational vector representations of the network nodes separately through matrix factorization and combines the k-order relational vector representations as the final representation. For a network G , the degree matrix D of the network is defined using the adjacency matrix S . The first-order transfer probability matrix is defined as follows:

$$A=D^{-1}S$$ (7)

where $A_{i,j}$ denotes the probability of transferring from $v_{i}$ to $v_{j}$ by one step.

Then using Skip-Gram and NCE (noise contrastive estimation) methods, for a transfer of order k , the model can be reduced to the decomposition problem with matrix $Y_{i,j}^{k}$ .

For a transfer of order k, the model is then reduced to a decomposition problem of matrix $Y_{i,j}^{k}$ by using the Skip-Gram and NCE (noise contrastive estimation) methods.

$$Y_{i,j}^{k}=W_{i}^{k}\cdot C_{j}^{k}=\log\left(\frac{A_{i,j}^{k}}{\sum_{t}A_{t,j}^{k}}\right)-\log\left(\beta \right)$$ (8)

where β= λ/N , λ is the number of negative samples and N is the number of edges in network G .

Node2vec designs a biased random wander over a scalable node neighborhood and explores different node neighborhoods by using breadth first search (BFS) and depth first search (DFS), and then inputs the resulting node sequences as sentences into the skip-gram model to learn node representations. For a random wandering sequence $v_{i-w},\ldots ,v_{i-1},v_{i},v_{i+1},\ldots ,v_{i+m}$ , centered on $v_{i}$ with windows size w . Node2vec uses the Skip-Gram algorithm to optimize the model.

$$\mathrm{Pr}\left(\left\{v_{i-w},\ldots ,v_{i+w}\right\}\backslash v_{i}|\Phi \left(v_{i}\right)\right)=\overset{i+w}{\underset{j=i-w,j\neq i}{\prod }}\backslash \mathrm{Pr}\left(v_{j}|\Phi \left(v_{i}\right)\right)$$ (9)

**FIGURE 3:**
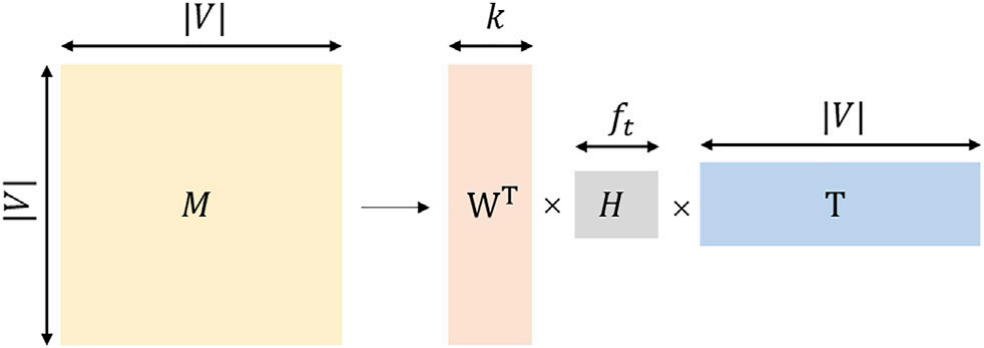
The Matrix factorization flow chart of TADW.

TADW utilizes the inductive matrix decomposition method based on integrating the network topology information and textual information. The Matrix factorization flow chart of TADW is shown in Figure 3.where V denotes the set of nodes, $W\in R^{k\times |V|}$ , $M\in R^{\left|V|\times |V\right|}$ , $H\in R^{k\times f_{t}}$ , and $T\in R^{f_{t}\times |V|}$ , and T is the text feature matrix. Therefore, the loss function of TADW as follows:

$$\underset{W,H}{\min}{\left\|M-W^{T}HT\right\|}_{F}^{2}+\frac{\lambda }{2}\left({\left\|W\right\|}_{F}^{2}+{\left\|H\right\|}_{F}^{2}\right)$$ (10)

These four state-of-the-art network representation learning methods can capture valuable information of the structure and intrinsic properties of the heterogeneous network. The learned representations of lncRNAs and mRNAs were further utilized to construct WAFNRLTG.

### Weighted Average Fusion Node Representation

The processes of fusing the node representations derived from the network representation learning methods (Grarep, LINE, TADW, and Node2vec) are described below. First, low-dimensional representations of lncRNAs and mRNAs were obtained from the heterogeneous network. Thus, the four representation vectors of nodes were obtained with different methods were $V_{1}$ , $V_{2}$ , $V_{3}$ and $V_{4}$ , and the corresponding prediction models were constructed based on these vectors. The AUC score of each model was calculated as the weighted value of the corresponding methods, they were labeled as $AUC_{1},\;AUC_{2},\;AUC_{3}$ and $AUC_{4}$ . To make full use of the information in node representation vectors and improve the generalization ability of the model, the new representation vector V was obtained by the weighted average fusion of the four representation vectors.

$$V=\frac{V_{1}*AUC_{1}+V_{2}*AUC_{2}+V_{3}*AUC_{3}+V_{4}*AUC_{4}}{AUC_{1}+AUC_{2}+AUC_{3}+AUC_{4}}$$ (11)

## Results

### Parameter and Experimental Settings

In this study, four network representation learning methods (GraRep, LINE, TADW, and Node2vec) were employed to obtain node representation vectors from the heterogeneous network.

First, we took the dimensions of the node representation vectors as the common parameter of these four methods. Tuning dimensions d , and the d -dimensional features that produced the best AUC were selected. The experimental results under node representation vectors of different dimensions are shown in Table 1.

**TABLE 1:** AUC scores of models with different dimension node representation vectors.

Method	d=16	d=32	d=64	d=128	d=256
GraRep	0.8725	0.8991	0.9095	0.9147	0.8957
LINE	0.8375	0.8939	0.9177	0.9123	0.8828
TADW	0.7974	0.8466	0.8803	0.8397	0.9002
Node2vec	0.8709	0.8978	0.9131	0.9099	0.9115

The other parameters of the network representation learning method are discussed in the following. GraRep has a parameter: k-step k , which indicates the k-step transfer matrix. Node2vec has four tunable parameters: number-walks *n*, indicates the number of random walks from each node; walk-length l , indicates the length of a random walk from each node; *p* and *q* control the probability of the random walk to the next node. The combination of number-walks *n* and walk-length *l* were considered, and the rest of the parameter was set as defaults. TADW has a parameter: λ , which controls the weight of the regularization term. In LINE, a parameter: Order o , denotes the order of proximity was considered. Above all, we adjusted different parameter values and adopt the optimal values which produced the best AUC scores. The parameter settings for various of the network representation learning method are shown in Table 2. For more details, please see Supplementary File S1.

**TABLE 2:** Parameters settings for network representation learning methods.

Method	Optimal parameter value
GraRep	d : 128, k : 4
LINE	d : 64, o : 3
TADW	d : 256, λ :0.1
Node2vec	d : 64, n : 20, l :80

### Comparison With Four Network Representation Learning Methods

In this section, we first constructed four models based on four representation network representation learning methods (Grarep, LINE, TADW, and Node2vec) and evaluated their effectiveness. In order to make full use of four network representation learning methods, the AUC scores of each method were adopted as weight values, and the four representation vectors were fused by the weighted average method. According to these constructed representation vectors, XGBoost classifier (Chen and Guestrin, 2016) was selected into WAFNRLTG.

**FIGURE 4:**
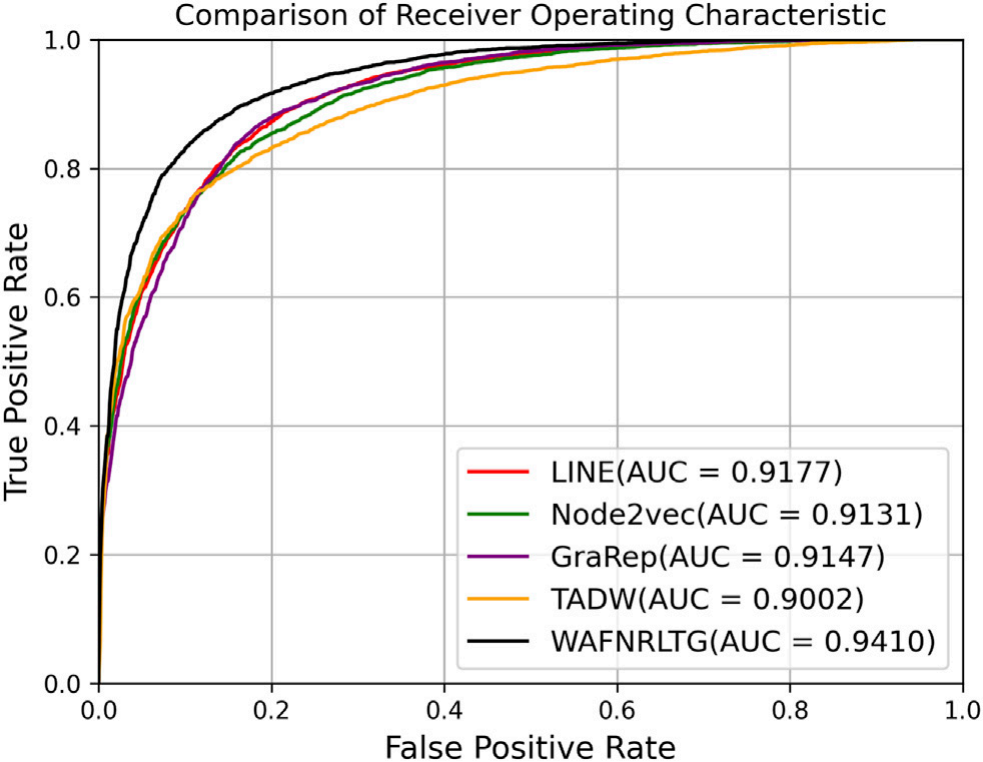
ROC curves of five models in five-fold cross-validations.

**FIGURE 5:**
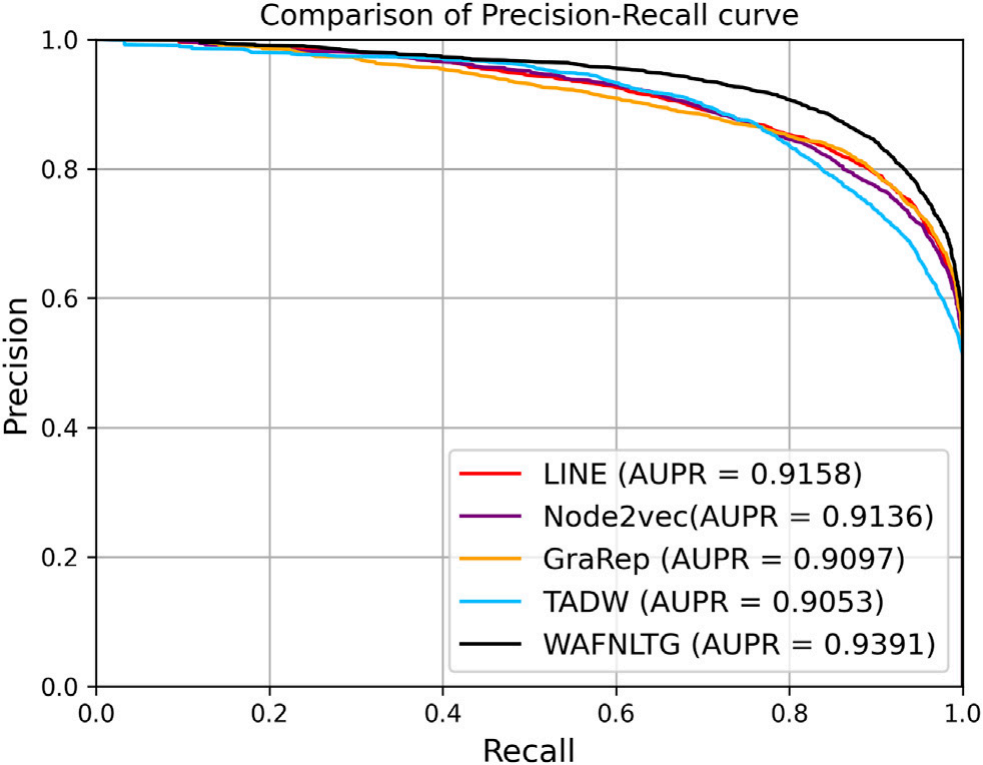
PR curves of five models in five-fold cross-validations.

Based on these four representations from the above network representation learning models, we subsequently fused them by the weighted average method to improve performance and generalization of WAFNRLTG. For assessing the prediction performance of WAFNRLTG, five-fold cross-validations experiments were used to evaluate the classification performances and the four network representation learning models. In our study, seven commonly metrics, namely Sensitivity (SN), Specificity (SP), Precision (PREC), Accuracy (ACC), Matthews correlation coefficient (MCC), AUC, and AUPR, were employed as evaluation metrics. ROC (receiver operating characteristic curve, ROC) and PR (Precision-Recall curve, PR) curves were plotted for showing the different performance of each model. The results of WAFNRLTG and the four network representation learning models are shown in Table 3. It can been seen from the table that WAFNRLTG achieves AUC score of 0.9410 and AUPR score of 9,391, which outperforms GraRep (AUC score: 0.9147; AUPR score: 0.9097), LINE (AUC score: 9,177; AUPR score: 0.9158), TADW (AUC score: 0.9002; AUPR score: 0.9053) and Node2vec (AUC score: 0.9131; AUPR score: 0.9136). ROC and PR curves are plotted for the five models to further display their different performances, Figure 4 shows five ROC curves and Figure 5 shows five PR curves.

**TABLE 3:** Performances of the five network representation learning models.

Method	Acc	Sen	Spec	Prec	MCC	AUC	AUPR
GraRep	0.8107	0.7920	0.8295	0.8229	0.6220	0.9147	0.9097
LINE	0.8358	0.8275	0.8441	0.8413	0.6718	0.9177	0.9158
TADW	0.8223	0.7798	0.8648	0.8523	0.6471	0.9002	0.9053
Node2vec	0.8305	0.8328	0.8282	0.8290	0.6611	0.9131	0.9136
WAFNRLTG	0.8683	0.8638	0.8728	0.8716	0.7362	0.9410	0.9391

For comparative analysis, we also adopted the other two fusion methods (concatenate and average) to fuse the four node representation vectors similar to the weighted average method. The experimental results of the three fusion methods are recorded in Table 4. As shown in Table 4, the weighted average fusion method outperforms other methods.

**TABLE 4:** The experimental results of the three fusion methods.

Method	ACC	SEN	SPEC	PREC	MCC	AUC	AUPR
Concatenate	0.86	0.8479	0.8722	0.8691	0.7203	0.9391	0.9307
Average	0.8515	0.8412	0.8618	0.8589	0.7033	0.9296	0.9277
Weighted average	0.8683	0.8638	0.8728	0.8716	0.7362	0.9410	0.9391

Moreover, the information extracted from heterogeneous networks by WAFNRLTG are brought to the subsequent work for predicting of lncRNA target genes.

In order to evaluate the generalization ability of WAFNRLTG, we applied it on the training dataset and the independent dataset. Experimental results are shown in Table 5. As exhibited from Table 5, the results on the independent dataset are comparable to the results on the training dataset. The experimental results on the independent dataset demonstrate that WAFNRLTG is a robust and reliable model for predicting potential lncRNA target genes.

**TABLE 5:** Performances of WAFNRLTG on the independent dataset and the training dataset.

Dataset	Acc	Sen	Spec	Prec	MCC	AUC	AUPR
independent dataset	0.8666	0.8567	0.8765	0.874	0.7333	0.9358	0.9350
training dataset	0.8683	0.8638	0.8728	0.8716	0.7362	0.9410	0.9391

### Comparison Among Different Classifiers

**FIGURE 6:**
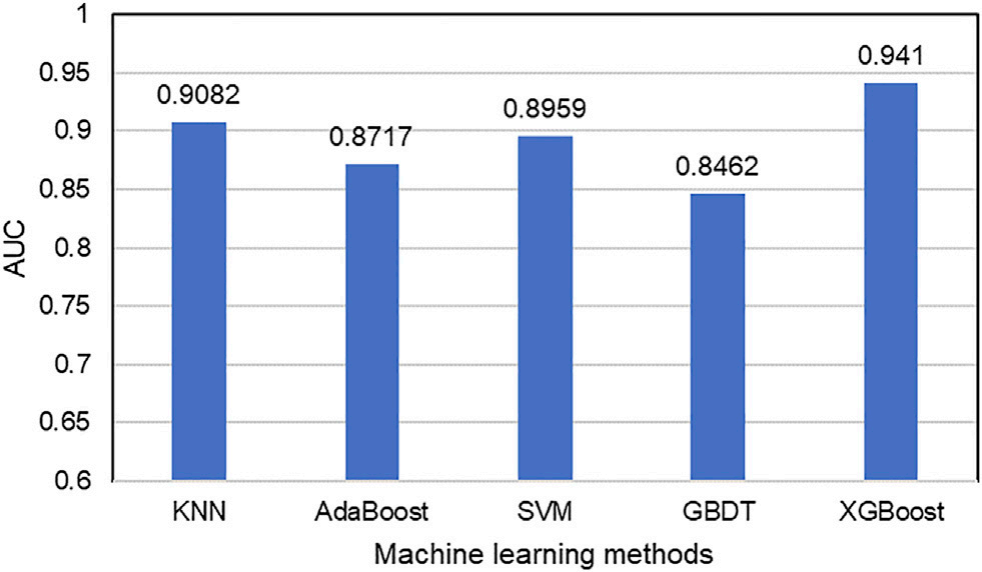
Comparisons of AUC scores of five classifiers.

After the acquisition of weighted averages fused representation vectors, we compared five different machine learning methods and selected the most appropriate one. These five classification methods include K Nearest Neighbor (KNN), AdaBoost (Freund and Schapire, 1997), Support Vector Machine (SVM), Gradient Boosting Decision Tree (GBDT) and XGBoost, which are well known to perform well on a variety of tasks. They were evaluated by five-fold cross-validations. Figure 6 shows their prediction performances. The AUC scores of KNN, AdaBoost, SVM, GBDT and XGBoost are 0.9082, 0.8717, 0.8959, 0.8462, and 0.9394, respectively. The detailed results of these five classifiers are shown in Table 6. From the analysis of above results, XGBoost model achieved the best performance.

**TABLE 6:** The detailed experimental results of the five classifiers.

Classifier	ACC	SEN	SPEC	PREC	MCC	AUC	AUPR
GBDT	0.8235	0.8037	0.8433	0.8369	0.6475	0.9082	0.9039
KNN	0.7197	0.7604	0.679	0.7037	0.4412	0.8012	0.8005
RF	0.785	0.7761	0.794	0.7907	0.5703	0.8718	0.8745
SVM	0.8063	0.784	0.8284	0.8204	0.6131	0.8959	0.8961
XGBoost	0.8683	0.8638	0.8728	0.8716	0.7362	0.9410	0.9391

### Case Studies

The main objective of this study is to screen potential target genes of lncRNAs and guide relevant researchers to explore novel target genes. For further evaluating the performance of WAFNRLTG model in practical applications, we selected three common lncRNAs (MALAT1, PVT1, and NEAT1) as case studies. The general processes of each of case studies were as following. First, all lncRNA-mRNA interactions from our dataset were utilized to construct the WAFNRLTG model. Then, the interactions between above three lncRNAs and the other mRNAs were adopted as the dataset in WAFNRLTG model. The model outputted the predicting scores of the lncRNA-mRNA pairs. Finally, the top ten mRNAs were selected for literature mining on PubMed. We found that four of the top ten mRNAs corresponding to MALAT1 and NEAT1 were validated in the literature, and three of the top ten mRNAs corresponding to PVT1 were validated in the literatures.

MALAT1 is one of the first lncRNA discovered that are associated with human diseases. Many studies have demonstrated that the abnormal expression of MALAT1 is closely related to cancer pathophysiology, and has the potential to be translated clinically. MALAT1 regulates cancer processes by interacting with molecules, such as proteins, RNAs and DNAs, and further alters different signal pathways. To demonstrate the ability of our model for predicting potential lncRNA target genes, we predicted the top ten mRNAs that interact with MALAT1, as shown in Table 7. With literature mining, we found that four mRNAs interact with MALAT1 and they can be used as the target genes of MALAT1. For example, abnormal expression of MALAT1 leads to reduced expression of TET2 thus causing neuronal damage, so there may be a targeting relationship between MALAT1 and TET2 (Li et al., 2021). The complete case study results of MALAT1 are available in Supplementary File S2.

**TABLE 7:** The predicting target genes in the top ten for MALAT1.

Rank	Score	Target genes	PMID
1	0.9952035	MYC	33312756
2	0.953083	MT-ND4L	Not found
3	0.952141	LRRC2	Not found
4	0.947306	TET2	33165916
5	0.92575	ECT2	27313681
6	0.923501	OR1M1	Not found
7	0.917459	ATXN2L	Not found
8	0.917261	TP73AS1	32714991
9	0.915255	NFIC	Not found
10	0.908282	FASTK	Not found

NEAT1 expression is upregulated in many human malignancies, such as lung, esophageal and gastric cancers. In order to demonstrate that WAFNRLTG is effective in predicting potential target genes of lncRNAs, we predicted the top 10 mRNAs associated with NEAT1, and the results are shown in Table 8. After literature mining, four of them were shown to be target genes of NEAT1. For example, it was found that MYC-regulated NEAT1 promoted diffuse large B-cell lymphoma (DLBCL) proliferation via the miR-34b-5p-GLI1 pathway, which could provide a novel therapeutic target for DLBCL (Qian et al., 2020). The complete case study results of NEAT1 are available in Supplementary File S3.

**TABLE 8:** The predicting target genes in the top ten for NEAT1.

Rank	Score	Target genes	PMID
1	0.975445	SUSD6	Not found
2	0.961867	CPN2	Not found
3	0.952019	PEX26	Not found
4	0.951388	WNT9A	Not found
5	0.942409	SRP19	Not found
6	0.931132	MYC	32206038
7	0.926551	OAS3	33138195
8	0.925127	CPSF6	22960638
9	0.924981	QSOX2	Not found
10	0.910639	TET2	33987091

Plasmacytoma variant translocation 1 (PVT1) is a newly discovered long non-coding RNA which preforms regulating functions as an oncogenic molecule in different cancers. In order to understand the functions of PVT1, WAFNRLTG predicted its target genes. We conducted a literature survey of the top ten mRNAs predicted by WAFNRLTG to interact with PVT1 and found that three mRNAs were verified to be its target genes, as shown in Table 9. For example, the abnormal expression of PVT1 affects the expression of NANOG and thus makes difference in the development of glioma (Gong et al., 2021). The complete case study results of PVT1 are available in Supplementary File S4.

**TABLE 9:** The predicting target genes in the top ten for PVT1.

Rank	Score	Target genes	PMID
1	0.971728	NANOG	34230224
2	0.9714675	GID4	Not found
3	0.970239	WNT9B	Not found
4	0.969168	TRPM3	Not found
5	0.966868	COL5A2	33750300
6	0.966181	RCC2	Not found
7	0.963472	RBM7	Not found
8	0.961825	WNT3A	32727463
9	0.957906	FAM101B	Not found
10	0.953643	CCDC115	Not found

## Discussion

LncRNAs and its target genes are involved in a variety of biological processes and are closely associated with serious human diseases. Predicting the potential lncRNA target genes can decipher complex biological mechanisms and reveal the functions of lncRNAs. In this paper, we firstly collected and processed multiple data from the multiple open databases, including lncRNA-mRNA interactions, lncRNA-miRNA interactions and mRNA-miRNA interactions. The lncRNA sequence similarity network and mRNA sequence similarity network were constructed based on sequence features. Then, we proposed a novel model, WAFNRLTG, to infer potential lncRNA target genes by integrating above data. In five-fold cross-validations on training and independent dataset, the experimental results demonstrated that WAFNRLTG obtained AUC scores (0.9410, 0.9350) and AUPR scores (0.9391, 0.9350). Three common lncRNAs (MALAT1, NEAT1, and PVT1) were introduced to WAFNRLTG model. Several target genes in the predicted results were found according to experimental PubMed literatures.

Although WAFNRLTG has achieved satisfactory results in predicting lncRNA target genes, it is still necessary to point out there are still limitations in our model. For example, the negative samples randomly selected from unknown lncRNA-mRNA interactions may have the chance of becoming positive samples. This would have an impact on the accuracy of WAFNRLTG. The information extracted from the heterogeneous network is not comprehensive, some valuable information may be neglected. With the accumulation of biological data, more interaction records would be introduced to enrich the heterogeneous network and improve the prediction ability of WAFNRLTG.

## Data Availability

The original contributions presented in the study are included in the article/Supplementary Material, further inquiries can be directed to the corresponding author.

## Supplementary Material

Supplementary File S1 The experimental details of WAFNRLTG.

Supplementary File S2 The prediction of MALAT1 target genes results of WAFNRLTG.

Supplementary File S3 The prediction of NEAT1 target genes results of WAFNRLTG.

Supplementary File S4 The prediction of PVT1 target genes results of WAFNRLTG.
