# Supplementary material for: WAFNRLTG: A Novel Model for Predicting LncRNA Target Genes Based on Weighted Average Fusion Network Representation Learning Method
Source: Front Cell Dev Biol. 2022 Jan 19;9:820342. doi: 10.3389/fcell.2021.820342 (PMC8807548; doi:10.3389/fcell.2021.820342)
Supplement: Supplementary file 2 [file Table1.DOCX]

**Experimental code details of WAFNRLTG**

**Part 1. Details of cross-validation experiments:**

Number of positive samples: 4529

Number of negative samples: 4529

5-fold cross validation:

Train Set Samples : Validation Set Samples = 7246 : 1812

All classifiers' parameters were set as defaults in XGBoost.

**Part 2. Results of cross-validation experiments:**

|  | ACC | SEN | SPEC | PREC | MCC | AUC |
| --- | --- | --- | --- | --- | --- | --- |
| Fold-1 | 0.883 | 0.8797 | 0.8863 | 0.8856 | 0.766 | 0.9509 |
| Fold-2 | 0.8515 | 0.8466 | 0.8565 | 0.8551 | 0.7031 | 0.929 |
| Fold-3 | 0.8598 | 0.8543 | 0.8653 | 0.8638 | 0.7197 | 0.9356 |
| Fold-4 | 0.8863 | 0.8874 | 0.8852 | 0.8855 | 0.7726 | 0.9518 |
| Fold-5 | 0.8608 | 0.8508 | 0.8707 | 0.8681 | 0.7217 | 0.9385 |
| Mean | 0.8683 | 0.8638 | 0.8728 | 0.8716 | 0.7366 | 0.9412 |

**Part 3. Python and packages versions for WAFNRLTG**

| **Python and Package Name** | **Python and Package Version** |
| --- | --- |
| Python | 3.7.x |
| networkx | 2.0 |
| numpy | 1.14 |
| pandas | 0.20.3 |
| scikit-learn | 0.24.2 |
| scipy | 0.19.1 |
| tensorflow | 1.15.0 |
| genism | 0.19.0 |

**Part 3. Results of parameters tuning for four network representation learning methods**

For the Grarep, we chose *k-step* in {1, 2, 3, 4}. The experimental results are shown in Table 1.

Table 1. The result of the selection of parameter *k-step*

|  | ACC | SEN | SPEC | PREC | MCC | AUC |
| --- | --- | --- | --- | --- | --- | --- |
| *k-step=*1 | 0.7737 | 0.743 | 0.8044 | 0.7919 | 0.5485 | 0.8582 |
| *k-step=*2 | 0.7987 | 0.7827 | 0.8147 | 0.8086 | 0.5978 | 0.884 |
| *k-step=*3 | 0.8262 | 0.8172 | 0.8353 | 0.8323 | 0.6526 | 0.9099 |
| *k-step=*4 | 0.8369 | 0.8251 | 0.8487 | 0.8452 | 0.6741 | 0.9148 |

In the LINE, we consider the *order* in {1, 2, 3}. The experimental results are shown in Table 2.

Table 2. The result of the selection of parameter *order*

|  | ACC | SEN | SPEC | PREC | MCC | AUC |
| --- | --- | --- | --- | --- | --- | --- |
| *order*=1 | 0.7952 | 0.7772 | 0.8132 | 0.8061 | 0.5909 | 0.8857 |
| *order* =2 | 0.6317 | 0.5856 | 0.6778 | 0.645 | 0.5646 | 0.6893 |
| *order* =3 | 0.8358 | 0.8275 | 0.8441 | 0.8413 | 0.6718 | 0.9177 |

For the two parameters of the Node2vec, *number-walks* in {10, 20, 30, 40} and *walk-lengths* in {20, 40, 60, 80, 100} are chosen. The experimental results are shown in Table 3 and Table 4.

Table 3. The result of the selection of parameter *number-walks*

|  | ACC | SEN | SPEC | PREC | MCC | AUC |
| --- | --- | --- | --- | --- | --- | --- |
| *number-walks* =10 | 0.7414 | 0.7286 | 0.7543 | 0.7479 | 0.4832 | 0.8321 |
| *number-walks* =20 | 0.8305 | 0.8328 | 0.8282 | 0.8290 | 0.6611 | 0.9131 |
| *number-walks* =30 | 0.8111 | 0.8026 | 0.8196 | 0.8167 | 0.6225 | 0.8968 |
| *number-walks* =40 | 0.7927 | 0.788 | 0.7973 | 0.7955 | 0.5854 | 0.8806 |

Table 4. The result of the selection of parameter *walk-lengths*

|  | ACC | SEN | SPEC | PREC | MCC | AUC |
| --- | --- | --- | --- | --- | --- | --- |
| *walk-lengths* =20 | 0.7737 | 0.7675 | 0.7799 | 0.7772 | 0.5475 | 0.8647 |
| *walk-lengths* =40 | 0.771 | 0.7651 | 0.777 | 0.7743 | 0.5423 | 0.8615 |
| *walk-lengths* =60 | 0.7719 | 0.7644 | 0.7794 | 0.7761 | 0.544 | 0.8592 |
| *walk-lengths* =80 | 0.8305 | 0.8328 | 0.8282 | 0.8290 | 0.6611 | 0.9131 |

For the TADW method, the *λ* was selected among {0.1, 0.2, 0.3, 0.4}. The experimental results are shown in Table 5.

Table 5. The result of the selection of parameter *λ*

|  | ACC | SEN | SPEC | PREC | MCC | AUC |
| --- | --- | --- | --- | --- | --- | --- |
| *λ*=0.1 | 0.8223 | 0.7798 | 0.8648 | 0.8523 | 0.6471 | 0.9002 |
| *λ*=0.2 | 0.7746 | 0.7419 | 0.8072 | 0.794 | 0.5506 | 0.8532 |
| *λ*=0.3 | 0.7755 | 0.7406 | 0.8103 | 0.796 | 0.5524 | 0.8517 |
| *λ*=0.4 | 0.7502 | 0.7081 | 0.7922 | 0.7731 | 0.5022 | 0.8274 |
